# Temporal Dynamics of Airborne Bacterial and Fungal Communities in Megacity Shanghai: Diel Asynchrony, Environmental Drivers, and Cold Front Perturbations

**DOI:** 10.3390/microorganisms14091975

**Published:** 2026-09-07

**Authors:** Jiaxin Wang, Ling Li, Yanyu Wang, Wenwen Sun, Tiantao Cheng

**Affiliations:** 1 Department of Atmospheric and Oceanic Sciences, Fudan University, Shanghai 200438, China; 24213020041@m.fudan.edu.cn; 2 Department of Environmental Science and Engineering, Fudan University, Shanghai 200438, China; liling@fudan.edu.cn; 3 Key Laboratory of Formation and Prevention of Urban Air Pollution Complex, Ministry of Ecology and Environment, Shanghai Academy of Environmental Sciences, Shanghai 200233, China; wangyy@saes.sh.cn; 4 Department of Research, Shanghai University of Medicine & Health Sciences Affiliated Zhoupu Hospital, Shanghai 201318, China; 5 Shanghai Key Laboratory of Ocean-Land-Atmosphere Boundary Dynamics and Climate Change, Fudan University, Shanghai 200438, China

**Keywords:** bioaerosols, community structure, environmental factors, diel variation, strong cold-air event

## Abstract

Atmospheric bacteria and fungi are key components of urban bioaerosols, and their community structures are closely related to urban air quality, bioaerosol concentration, and environmental driving factors. However, the temporal dynamics and environmental drivers of bacterial and fungal community structures remain poorly characterized in megacities such as Shanghai, China. From November 2024 to November 2025, we conducted a year-long, cross-seasonal bioaerosol sampling and high-throughput DNA sequencing campaign to investigate the seasonal and diel variations, environmental covariation, and meteorological responses of atmospheric bacteria and fungi in Shanghai. The results show that seasonal transitions cannot significantly alter the α-diversity of bacterial or fungal communities, but significantly shift their community compositions, exhibiting a stronger differentiation for fungi than bacteria (PERMANOVA *R*^2^ = 0.133 vs. 0.054, *p* = 0.001). At the diel scale, bacterial communities exhibit weak diurnal variation, whereas fungal communities display significantly higher nocturnal Chao1 richness and Shannon diversity, along with distinct compositional separation. *Cladosporium* and *Alternaria* are enriched during the day, while saprotrophic basidiomycetes such as *Irpex* and *Trametes* are more abundant at night. Fungal composition covaries most strongly with meteorological gradients governed by boundary layer height, clear-sky surface downward solar radiation, temperature, relative humidity, atmospheric pressure and visibility, whereas bacterial–environment associations are weaker. The Relative Exposure-Relevant Proportion (RERP), dominated by *Cladosporium* and *Alternaria*, is higher during daytime and negatively associated with relative humidity (*β* = −0.23, *q* = 0.001). During a strong cold-air event, bacterial communities shifted rapidly following immediate air mass replacement and high wind shear before stabilizing, whereas fungal communities underwent a more gradual and cumulative turnover. The RERP also elevated during this event, accompanied by a greater contribution from *Talaromyces*. These findings reveal contrasting dynamics between airborne bacteria and fungi under both background and transient synoptic disturbances, providing crucial observational insights for bioaerosol modeling and risk prediction in urban environments.

## 1. Introduction

Bioaerosol is one important carrier to connect terrestrial and aquatic surfaces with the atmosphere to cross layer material and energy exchange in the Earth system. Bacteria and fungi represent major components of bioaerosols due to their high atmospheric abundance and ubiquitous global distribution [1,2]. Every year, large quantities of bacterial cells and fungal spores, about millions of tons, are emitted into the atmosphere from soil, sea spray, plant surfaces, and anthropogenic activities worldwide [3]. These biological particles play important roles in atmospheric chemistry, cloud formation acting as cloud condensation nuclei (CCN) or ice-nucleating particles (IN), regional climate dynamics and biogeochemical cycles upon deposition [4,5]. Although certain airborne genera comprise recognized opportunistic pathogens or allergens, marker gene sequencing alone provides relative structural profiles rather than absolute viability, inhalation dose, or direct health risk [6,7]. Systematically deciphering the temporal variations in and environmental drivers of airborne bacteria and fungi is therefore essential for realistic bioaerosol modeling and health impact assessments.

Atmospheric bacterial and fungal communities vary across space and time in association with environmental status, influenced by a combination of temperature, relative humidity, pressure, and air pollutions [8,9]. Recent studies further demonstrate that these patterns emerge from multiple interacting processes, including meteorological forcing on the bioaerosol life cycle (i.e., emission, transport, and persistence), predictable spatiotemporal turnover of fungal communities, and taxon-specific adaptations to atmospheric stressors [8,9,10]. While seasonal surveys and episodic pollution events have shed light on the shifts in airborne microbial diversity and composition [11,12,13], the fine-scale diel patterns of urban bioaerosols remain insufficiently understood. Previous studies reported day–night variation in bioaerosol concentrations and community composition, yet the underlying physical drivers and release mechanisms remain poorly constrained [14,15]. Fungal release can depend on humidity and spore release mode [16,17]. In contrast, airborne bacteria are frequently attached to coarse particulate matter, undergoing resuspension and regional transport driven by anthropogenic turbulence and surface winds [18,19]. These mechanistic discrepancies give rise to asynchronous temporal patterns between bacterial and fungal communities.

Meteorological variables and pollutants have repeatedly been shown to be closely associated with airborne microbial communities [20]. However, the robustness of these relationships exhibits a strong size-dependence, reflecting the stark divergence in bacterial and fungal community structures between fine and coarse aerosols [21,22,23]. In particular, a substantial proportion of airborne fungal spores occurs in the coarse particle size range [24]. As a widely standardized aerosol fraction in urban air quality and atmospheric chemistry studies, PM_2.5_ contains abundant secondary inorganic ions, carbonaceous components, and trace elements that reflect important atmospheric processes, including combustion-related emissions, traffic pollution, secondary aerosol formation, and regional transport [25]. Compared with PM_2.5–10_, which is generally more strongly influenced by mechanically generated materials such as mineral dust, road dust, and resuspended particles, PM_2.5_ provides a more informative measure of secondary aerosol formation, combustion-related emissions, and regional transport [26,27]. These measurements therefore provide complementary information on the evolving urban particulate pollution background. Despite the recognized importance of meteorology, gaseous pollutants, and particulate chemical conditions, integrated assessments of how these environmental dimensions covary with the temporal dynamics of airborne bacterial and fungal communities remain limited.

Near-surface bioaerosol assemblages reflect the interplay among local biological emissions, carrier particle dynamics, planetary boundary layer (PBL) mixing, and regional long-range transport [28,29]. Local vegetation and anthropogenic activities define the baseline biogenic emissions [30], whereas synoptic transport and mineral dust events import foreign microbial signatures from distant origin regions [31,32,33]. Abrupt synoptic perturbations—such as strong cold-air passages—rapidly alter thermal stratification, wind shear, air mass origins, and aerosol chemical processing. Because these physical and chemical drivers are tightly coupled, disentangling the distinct impact of individual meteorological drivers represents a major analytical challenge [12,34,35]. Observations at high latitudes also show that the concentrations of bacteria, pollen and fungal spores and their proportion in total bioaerosols differ between cold–wet and warm–dry conditions [36]. Moreover, while bioaerosol responses to haze, dust storms, and precipitation have been extensively documented, microbial community reorganization during rapid cold front transitions in coastal megacities remains less studied.

To address these gaps, we conducted a year-long cross-seasonal field campaign with high-resolution daytime/nighttime sampling and high-throughput marker gene sequencing in urban Shanghai. The main objectives of this study are three-fold: to elucidate the seasonal and diel variation patterns of airborne bacterial and fungal communities; to quantify community-level environmental associations across integrated meteorological and gaseous pollutants; and to evaluate exposure-relevant fungal dynamics (RERP) alongside the immediate versus lagged community restructuring triggered by a strong cold front passage. Overall, this work delivers comprehensive observational insights into the environmental coupling of urban bioaerosols under both baseline and disturbed synoptic conditions.

## 2. Data and Methods

### 2.1. Study Site and Sample Collection

Sampling was conducted at the Jiangwan Campus of Fudan University in Shanghai, a coastal megacity characterized by a typical subtropical monsoon climate. The sampler was positioned on a rooftop (31.34° N, 121.51° E) approximately 20 m above ground level. This height represents the urban canopy while reducing direct disturbance from surface dust and nearby ground-level activities. Sampling was conducted from November 2024 to November 2025, with seven consecutive days per month, with suspensions during heavy precipitation events to avoid rain-out wash-off artifacts. Specific retained sampling dates and the corresponding daytime/nighttime sample counts for each campaign are provided in Table S1.

Bioaerosols were collected using a high-flow intelligent microbial air sampler (ZR-2000, Qingdao Zhongrui Intelligent Instrument Co., Ltd., Qingdao, China) operating at 28.3 L/min. Particle size bins and classification parameters are listed in Table S2. Aerosols were collected on sterile 0.22 μm mixed cellulose ester filters. Filters from all size bins were combined before DNA extraction, so size-resolved communities were not analyzed. Daytime (08:00–19:30, UTC+8) and nighttime (20:00–07:30) samples were collected separately for 11.5 h. Before use, filters were rinsed in ultrapure water for 30 s, wrapped in aluminum foil, autoclaved at 121 °C for 30 min, and dried at 80 °C for 2 h. After sampling, filters were refrigerated during transport, transferred aseptically into sterile tubes, and stored at −80 °C. Quality assurance and quality control included flow calibration before and after sampling (within ±5%), aseptic cleaning, and field blanks. Environmental samples and blanks underwent the same DNA extraction and PCR procedures (Text S1.1). Field blanks yielded no visible PCR amplicons and fell below library construction thresholds, confirming negligible background contamination.

Fine particulate matter (PM_2.5_) was collected concurrently on 203 × 254 mm quartz fiber filters (Munktell MK360, Munktell & Filtrak, Falun, Sweden) using a high-volume sampler (TH-1000H, Tianhong, Suzhou, China) at 1.05 m^3^/min. To remove organic residues, filters were baked at 450 °C for 4 h before sampling. Mass concentration was determined gravimetrically using a microbalance (Sartorius MSA225S-1CE-DU, Sartorius AG, Göttingen, Germany) after 24 h equilibration under a controlled temperature (25 °C) and relative humidity (49%).

### 2.2. Sample Processing and Environmental Data

#### 2.2.1. DNA Extraction, Amplification and High-Throughput Sequencing

Total genomic DNA was extracted from aerosol filters with the MJ Water DNA Extraction Kit (Shanghai Majorbio Bio-pharm Technology Co., Ltd., Shanghai, China) according to the manufacturer’s instructions. Extracted DNA was aliquoted and stored at −80 °C. The bacterial V3-V4 region of the 16S rRNA gene was amplified with barcoded primers 338F (5′-ACTCCTACGGGAGGCAGCAG-3′) and 806R (5′-GGACTACHVGGGTWTCTAAT-3′) [21]. The fungal ITS1 region was amplified with barcoded primers ITS1F (5′-CTTGGTCATTTAGAGGAAGTAA-3′) and ITS2R (5′-GCTGCGTTCTTCATCGATGC-3′) [37,38]. Qualified amplicons were verified by 2% agarose gel electrophoresis, gel-purified, and quantified with a Qubit 4.0 fluorometer. Libraries were prepared with the NEXTFLEX Rapid DNA-Seq Kit (Bioo Scientific, Austin, TX, USA) and sequenced with paired-end reads on an Illumina NextSeq 2000 platform (San Diego, CA, USA).

Raw reads were quality-filtered, merged, and clustered into operational taxonomic units (OTUs) at 97% sequence similarity. Representative bacterial and fungal sequences were classified against the SILVA and UNITE databases, respectively. Core bioinformatic processing and initial statistical analyses were performed on the Majorbio Cloud Platform (https://cloud.majorbio.com, assessed on 15 June 2026). Detailed laboratory procedures and bioinformatic parameters are provided in Text S1.2.

#### 2.2.2. Measurements

PM_2.5_ chemical measurements characterized the bulk particulate background at the sampling site, not the surface chemistry of individual biological particles. For water-soluble ions, two pairs of 2 cm^2^ filter subsamples were sonicated in 10 mL ultrapure water for 30 min. Extracts were filtered through 0.22 μm nylon membranes and analyzed by ion chromatography (Dionex ICS-3000, Thermo Fisher Scientific, Sunnyvale, CA, USA). Nine ions were measured, including Na^+^, NH_4_^+^, K^+^, Mg^2+^, Ca^2+^, Cl^−^, NO_3_^−^, NO_2_^−^ and SO_4_^2−^. Organic carbon (OC) and elemental carbon (EC) were measured with a thermal–optical carbon analyzer (Sunset Laboratory, Hillsborough, NC, USA). Samples were heated sequentially in inert He and oxidizing He/O_2_ atmospheres, with laser transmission used to correct for pyrolyzed organic carbon. For metals, 3.0 cm^2^ filter subsamples were digested with 10 mL HNO_3_/HCl (1:3, *v*/*v*) at 200 °C for 15 min in an HG08Z-10 microwave digestion system. Digests were redissolved in 10 mL of 0.5% HNO_3_ and diluted to 50.0 mL. Li, Al, Ti, V, Cr, Mn, Fe, Ni, Cu, Zn, Sr, Mo, Cd, Sb, Ba and Pb were quantified by inductively coupled plasma mass spectrometry (ICP-MS). Method blanks, field blanks and certified reference materials accompanied each batch. Values below the method detection limit were replaced with one-half of the limit.

Local meteorological variables and conventional air pollutants were measured continuously at the Fudan University Atmospheric Environment Superstation, which was co-located with the bioaerosol sampler. SO_2_, NOx, CO and O_3_ were monitored with Thermo Scientific 43i-TLE, 42i-TL, 48i-TLE and 49i analyzers (Franklin, MA, USA), respectively. Temperature (T), relative humidity (RH), wind speed (WS), wind direction (WD), pressure (P), and visibility (Vis) were recorded with a Vaisala WXT536 automatic weather station (Vantaa, Finland). ERA5 provided clear-sky surface downward solar radiation (SSRDC) and boundary layer height (BLH) at 0.25° spatial and 1 h temporal resolution (https://cds.climate.copernicus.eu). Although the ERA5 variables provide a macroscopic view of the regional radiation and boundary layer, they cannot resolve localized microscale turbulent processes at the observation site.

### 2.3. Statistical Analyses

#### 2.3.1. Community Diversity and Differential Abundance

Cold and warm seasons were defined using fixed half-year periods commonly applied in East Asian studies: November–April and May–October, respectively. Samples collected during the strong cold-air event in November 2025 were excluded from the background seasonal analysis. The final cold- and warm-season groups had equal sample sizes. Diel analyses used paired day and night samples. Shannon diversity and Chao1 richness were calculated in mothur v1.48.0. Differences between seasons were evaluated using the Mann–Whitney U test, while paired diel variations were assessed via the Wilcoxon signed-rank test. Beta diversity was calculated based on Bray–Curtis dissimilarity matrices. To determine significant shifts in community composition across seasons, permutational multivariate analysis of variance (PERMANOVA; 999 permutations) was conducted, accompanied by PERMDISP to verify the homogeneity of multivariate dispersions. For assessing diel community changes, PERMANOVA permutations were strictly constrained within paired sampling dates.

Genus-level differences were evaluated separately by season and diel period. To reduce instability from rare taxa, seasonal tests included genera with mean relative abundance above 1% across samples. Mann–Whitney U tests compared cold and warm seasons, and the warm-minus-cold difference indicated the enrichment direction. Diel tests included genera with mean relative abundance above 1% among paired samples. Paired Wilcoxon signed-rank tests compared day and night, and the night-minus-day difference indicated the enrichment direction. *p* values were adjusted separately for bacteria and fungi using the Benjamini–Hochberg false discovery rate (BH-FDR); *q* < 0.05 indicated statistical support. Core bioinformatic processing used the Majorbio Cloud Platform. Data cleaning, statistical tests and visualization were conducted in Python v3.12.7 and R v4.3.1.

#### 2.3.2. Analysis of Environmental Associations

We constructed four community response matrices, that is, all bacterial genera, dominant bacterial genera, all fungal genera and dominant fungal genera. Dominant genera had a mean relative abundance above 1% across samples. Genus-level relative abundance matrices were Hellinger-transformed before constrained ordination and distance calculation to reduce the influence of zeros and sparsity. Environmental predictors were grouped into meteorology and gaseous pollutants. As outlined in the Introduction, PM_2.5_ chemical components were used as ambient fine-particle chemical background indicators rather than as direct effectors. Accordingly, they were excluded from community-level models. Principal component analysis (PCA) was applied separately to each module. PC1 was retained for every module, and PC2 was also retained when it explained at least 15% of the variance. These PCA axes were used as predictors. Module composition, variance inflation factors (VIFs) and selection criteria are reported in Table S3.

Distance-based redundancy analysis (db-RDA) of Hellinger-transformed Bray–Curtis dissimilarities was used to estimate the overall association between the environmental matrix and community composition. Conditional db-RDA models controlling for month were fitted as a robustness check, and significance was assessed with 999 permutations. Primary interpretation was limited to matrices for which the full model showed stable statistical support. Partial RDA controlling for month then estimated the contribution associated with each environmental module. Aggregated boosted tree (ABT) models provided a complementary assessment of predictive importance, using PCoA1 from the same dissimilarity matrix as the response [39,40]. The ABT predictor set was restricted to Meteorology PC1, Meteorology PC2, Pollutants PC1, and Pollutants PC2. Models used five-fold cross-validation stratified by sampling date and repeated 50 times (n_estimators = 160, learning_rate = 0.035, max_depth = 2, min_samples_leaf = 4, subsample = 0.75). Model performance was summarized using the mean, standard deviation, and 95% confidence intervals of the cross-validated *R^2^*, which were subsequently compared against a baseline established by a response permutation null model. Predictor importance was the mean decline in test fold *R^2^* after permutation, while negative values were treated as no positive predictive contribution. The bacterial models did not pass the robustness and predictive performance checks. The ABT results are therefore shown only for the all-genus and dominant-genus fungal matrices.

#### 2.3.3. Definition of Relative Exposure-Relevant Proportion

Due to the inability of 16S rRNA annotation to resolve environmental, commensal, and pathogenic species at the genus level, a bacterial exposure indicator was excluded from this analysis. Instead, we calculated the Relative Exposure-Relevant Proportion (RERP) for fungal genera with documented allergenic or opportunistic pathogenic potential. Candidate genera were compiled from bioaerosol studies and intersected with genera detected here [41,42,43,44]. The primary set retained genera that met at least one criterion: occurrence frequency ≥ 10%, mean relative abundance ≥ 0.1%, or ranking among the 20 most abundant genera. For the samples, the RERP was the sum of the relative abundances of selected genera:

(1) $$RERPs=\sum_{g\in G}{RA}_{g,s}$$

where G is the selected set of fungal genera, and $RA_{g,s}$ is the relative abundance of genus g in the samples. The RERP was log-transformed after adding a small constant to accommodate zero values:

(2) $${log}_{10}RERPs={log}_{10}(RERPs+\varepsilon_{R})$$

where $\varepsilon_{R}$ was half the smallest nonzero RERP value. To address compositional closure, individual target genera abundances were also transformed by the centered log ratio (CLR). Statistical tests used ${log}_{10}RERP$ or $CLR_{g,s}$ as response variables. Because the RERP is derived from sequencing-based relative abundance, it does not reflect absolute inhaled dose, airborne spore concentration, allergen exposure, biological viability, or direct health risk. We first compared the RERP between cold and warm seasons. Environmental models used month, rather than the broad seasonal grouping, as the temporal fixed effect because environmental variables varied strongly among months. This adjustment reduced confounding by month-specific phenology, pollution and meteorology. We fitted single-predictor linear mixed-effects models with standardized environmental variables while adjusting for month and diel period, following de Groot et al. [10]:

(3) $$Y_{s}=\beta_{0}+\beta_{1}E_{s}+\beta_{2}{Diel}_{s}+\beta_{3}{Month}_{s}+u_{s}+\varepsilon_{s}$$

where $Y_{s}$ is ${log}_{10}RERP$ or $CLR_{g,s}$, and *E_s_* is a z-standardized environmental predictor. Diel_s_ and Month_s_ are categorical fixed effects, $u_{s}$ is a random intercept for the paired sampling unit, and $\varepsilon_{s}$ is the residual error. Each model contained one focal environmental predictor to reduce coefficient instability from collinearity. Environmental predictors were divided a priori into two groups for analytical purposes. The core group—nine meteorological variables and four gaseous pollutants—served as the primary predictors in community/genus-level models. The supplementary group comprised water-soluble ions, OC, EC, and trace metals; these variables were analyzed separately and interpreted only as indicators of the contemporaneous fine-particle chemical environment. Outputs included standardized coefficients (*β*), *p* values, and Benjamini–Hochberg-adjusted *q* values. Figures show only associations with *q* < 0.05 and include separate descriptive fits for day and night. Because environmental variables were correlated, coefficients were interpreted as conditional associations rather than independent causal effects.

#### 2.3.4. Air Mass Trajectory Analysis

Potential air mass transport histories were assessed with 72 h backward trajectories from the HYSPLIT model. Meteorological input came from the Global Data Assimilation System at 1° × 1° resolution. Trajectories arrived at the Shanghai site (31.34° N, 121.51° E) at 500 m above ground level. This height was chosen to represent regional transport in the upper near-surface boundary layer. Coordinates were recorded hourly. Two trajectories were initialized each day at 00:00 and 12:00 UTC to approximate the transport backgrounds of the two sampling periods.

Trajectories were grouped by the synoptic stage assigned to each sampling date, such as pre-frontal, cold high-pressure and post-frontal. These groups describe transport backgrounds during the evolving weather system. They were not derived through statistical clustering and thus do not represent mathematically discrete trajectory groups.

## 3. Results and Discussion

### 3.1. Temporal Patterns of Urban Airborne Microbial Communities

#### 3.1.1. Differences Between Cold and Warm Seasons

Bacterial and fungal communities showed similar alpha diversity in cold and warm seasons but different community composition. Seasonal differences in α-diversity were generally weak. For bacteria, the Shannon index was comparable between the cold and warm seasons (4.254 ± 0.984 vs. 4.330 ± 0.824), whereas Chao1 richness was numerically higher in the cold season (443.17 ± 131.25) than in the warm season (384.84 ± 121.65). For fungi, neither Shannon diversity (2.850 ± 0.489 vs. 3.003 ± 0.594) nor Chao1 richness (115.27 ± 72.48 vs. 142.81 ± 101.40) differed significantly between the cold and warm seasons. Detailed seasonal α-diversity statistics are provided in Table S4. Alpha diversity metrics, specifically Shannon diversity and Chao1 richness, exhibited no significant seasonal variation for either microbial group, with *q* values ranging from 0.313 to 0.859 (all *q* > 0.05; Table S5). Previous research similarly indicates that airborne bacterial composition is highly responsive to environmental variations, even when overall alpha diversity remains stable [45]. The seasonal contrast at this site therefore reflected changes in the relative composition of taxa rather than overall richness or evenness.

**Figure 1:**
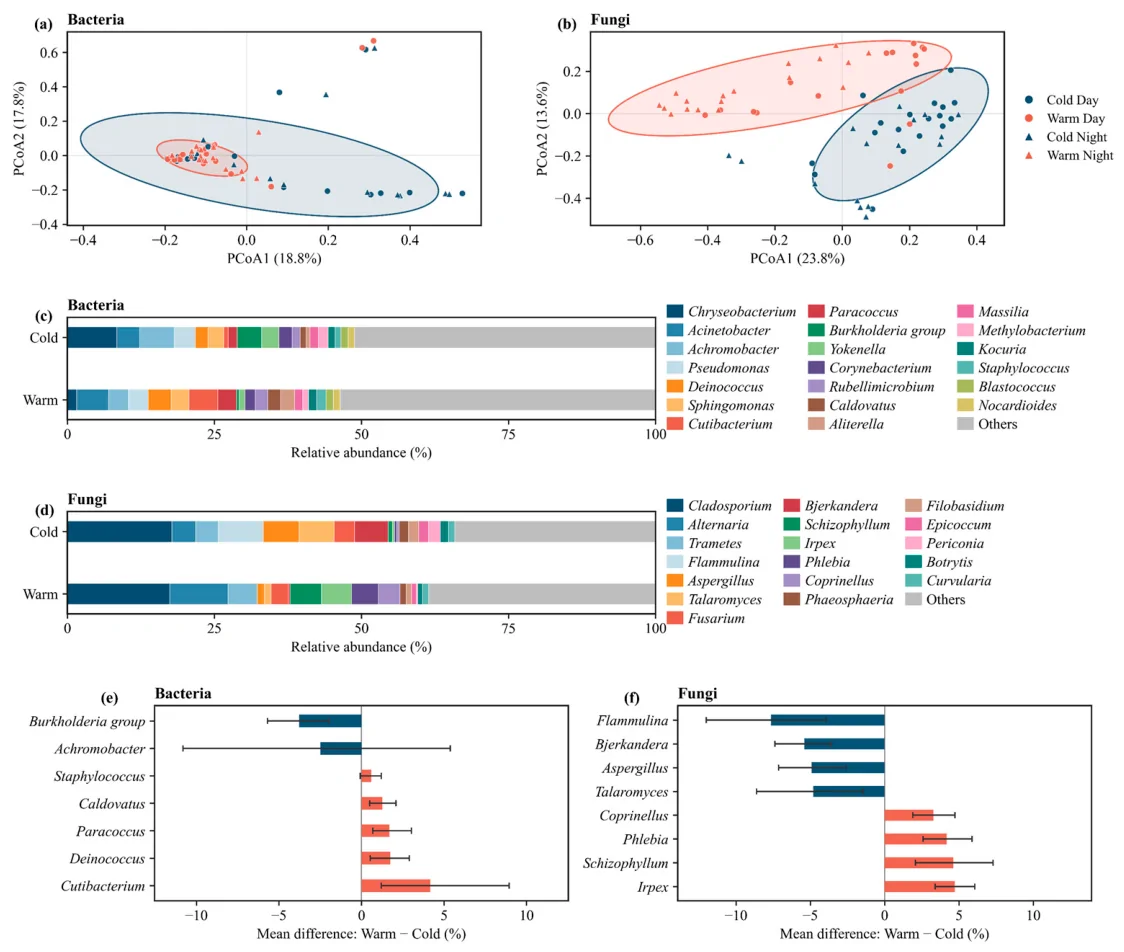
Seasonal differences in urban airborne bacterial and fungal communities. Bray–Curtis PCoA for bacteria (**a**) and fungi (**b**) in cold and warm seasons; points are samples, colors indicate season, and shapes indicate day/night. Mean relative abundances of dominant bacterial (**c**) and fungal (**d**) genera; genera < 1% are grouped as Others. (**e**,**f**) Warm-minus-cold differences for significant genera, in percentage points; error bars show bootstrap 95% CIs. Blue and red denote cold- and warm-season enrichment. Mann–Whitney U tests with BH-FDR correction.

PCoA and PERMANOVA indicated significant compositional differences between cold and warm seasons for both bacteria and fungi (Figure 1a,b; Table S5). PERMDISP was not significant, suggesting that these results were not explained by unequal within-group dispersion. Fungal samples separated more clearly along PCoA1, which is consistent with a stronger seasonal compositional signal in this dataset. Seasonality has been widely reported for airborne microbial communities, particularly fungi [9,46]. The observed patterns may reflect seasonal changes in vegetation, soil inputs, biological emissions and regional transport [9,11].

Dominant bacterial genera also differed between seasons. Cold-season samples contained high relative abundances of *Chryseobacterium* (8.40%), *Achromobacter* (5.86%), and *Burkholderia*–*Caballeronia*–*Paraburkholderia* (4.15%) groups, and the *Achromobacter* and *Burkholderia* groups were significantly enriched (*q* < 0.05). In contrast, warm-season samples were dominated by *Acinetobacter* (5.42%), *Cutibacterium* (4.91%), and *Deinococcus* (3.86%), with *Cutibacterium*, *Deinococcus*, *Paracoccus*, *Caldovatus*, and *Staphylococcus* showing significant enrichment (*q* < 0.05). The relative enrichment of human-associated *Cutibacterium* and *Staphylococcus* may reflect seasonal changes in occupancy-related emissions, ventilation, or boundary layer mixing [47,48]. The enrichment of stress-tolerant *Deinococcus* is consistent with greater solar radiation and desiccation during warmer periods [49]. The *Paracoccus* pattern may also covary with resuspended urban dust and nitrogen-rich particles [50]. These explanations remain tentative because sources were not measured directly.

Fungal communities showed a stronger seasonal turnover. *Cladosporium* remained abundant in both seasons (approximately 17–18%). Cold-season samples contained more *Flammulina* (7.64%) and *Aspergillus* (6.12%), with significant enrichment of *Flammulina*, *Aspergillus*, *Talaromyces* and *Bjerkandera*. Meanwhile, warm-season samples contained more *Alternaria* (9.86%) and *Schizophyllum* (5.29%), with significant enrichment of *Schizophyllum*, *Irpex*, *Phlebia* and *Coprinellus* (*q* < 0.05). These patterns are consistent with seasonal changes in temperature, humidity, vegetation and decomposing substrates, although the contributing sources were not resolved here [11]. Although the genus *Alternaria* comprises known allergenic species, its relative abundance cannot serve as a direct measure of absolute airborne concentration or actual human exposure [17]. Collectively, seasonal differences manifested mainly as taxonomic replacement among dominant groups, with fungi exhibiting a notably stronger compositional shift than bacteria.

#### 3.1.2. Diel Variation

Bacterial Shannon diversity was 4.222 during the day and 4.168 at night, while bacterial Chao1 richness was 420.2 and 392.4, respectively (Table S4). Diel comparisons provided a finer-scale resolution of temporal dynamics; however, bacterial alpha diversity and overall community composition exhibited no significant diurnal variation (*R^2^* = 0.017, *p* = 0.189; Table S5). This weak diel signal may reflect continuous particle inputs, resuspension, and mixing from several sources [18,19]. Daytime samples contained more *Achromobacter* (8.59%), *Chryseobacterium* (5.14%) and *Acinetobacter* (3.87%), whereas nighttime samples contained more *Acinetobacter* (5.62%), *Chryseobacterium* (5.57%) and *Cutibacterium* (4.57%). No bacterial genus remained significant after FDR correction, so these differences are treated as descriptive trends.

**Figure 2:**
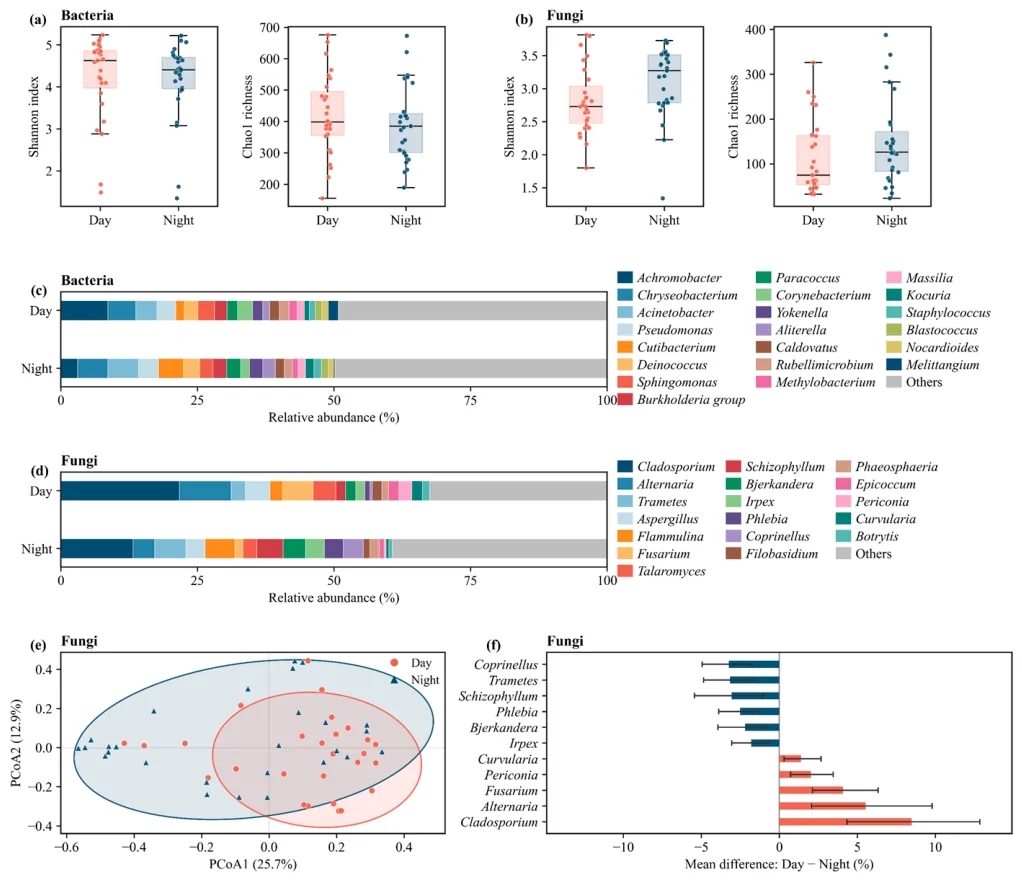
Stronger diel responses of fungal diversity, composition, and dominant genera. (**a**,**b**) Shannon diversity and Chao1 richness in paired day/night samples; boxes show medians and interquartile ranges, and points are samples. (**c**,**d**) Mean relative abundances of dominant bacterial (**c**) and fungal (**d**) genera; genera < 1% are grouped as Others. (**e**) Fungal Bray–Curtis PCoA by diel period. (**f**) Night-minus-day differences for significant fungal genera, in percentage points; error bars show bootstrap 95% CIs. Blue and red denote day and night enrichment. Paired Wilcoxon tests with BH-FDR correction.

Fungal Chao1 richness and Shannon diversity were higher at night (Table S4), and community composition differed between day and night (pseudo-F = 3.288, *R^2^* = 0.059, *p* = 0.001; Figure 2d,f). Daytime-enriched genera primarily included *Cladosporium* (21.69% vs. 13.21% at night), *Alternaria* (9.48% vs. 3.95%), and *Fusarium* (5.56% vs. 1.47%), alongside *Periconia* (2.36% vs. 0.33%) and *Curvularia* (1.89% vs. 0.50%). Conversely, nighttime communities were characterized by the enrichment of basidiomycetes such as *Trametes* (5.75% vs. 2.60% during the day), *Schizophyllum* (4.81% vs. 1.76%), *Bjerkandera* (4.11% vs. 1.90%), *Irpex* (3.47% vs. 1.68%), *Phlebia* (3.44% vs. 0.92%), and *Coprinellus* (3.67% vs. 0.43%). The daytime enrichment of *Cladosporium* and *Alternaria* aligns with dry spore release mechanisms typically triggered by warmer, drier, and more turbulent atmospheric conditions [51]. In contrast, the nocturnal peak of basidiomycetes likely reflects humidity-dependent spore discharge, reduced atmospheric dispersion, or inputs from local vegetation and leaf litter [52,53,54,55]. These nocturnal increases in wood decay fungi and associated tracers are consistent with previous observations [56,57,58]. However, to definitively disentangle these processes, direct measurements of local spore emission and source activities are required.

### 3.2. Environmental Associations Under Background Conditions

#### 3.2.1. Community-Level Multi-Module Analysis

We first assessed community covariation with meteorological variables and gaseous pollutants. These environmental variables exhibited substantial variability across both monthly and diel timescales (Table S6; Figure S1). The first two axes of overall environmental PCA explained 23.3% and 13.4% of the variance, respectively. Daytime conditions were associated with higher O_3_, temperature, wind speed, boundary layer height, and clear-sky surface downward solar radiation. Nighttime conditions were associated with higher relative humidity and NO_2_. This diurnal separation aligns with daytime boundary layer development and photochemical activity, as well as nocturnal surface accumulation [2,11,14,15].

**Figure 3:**
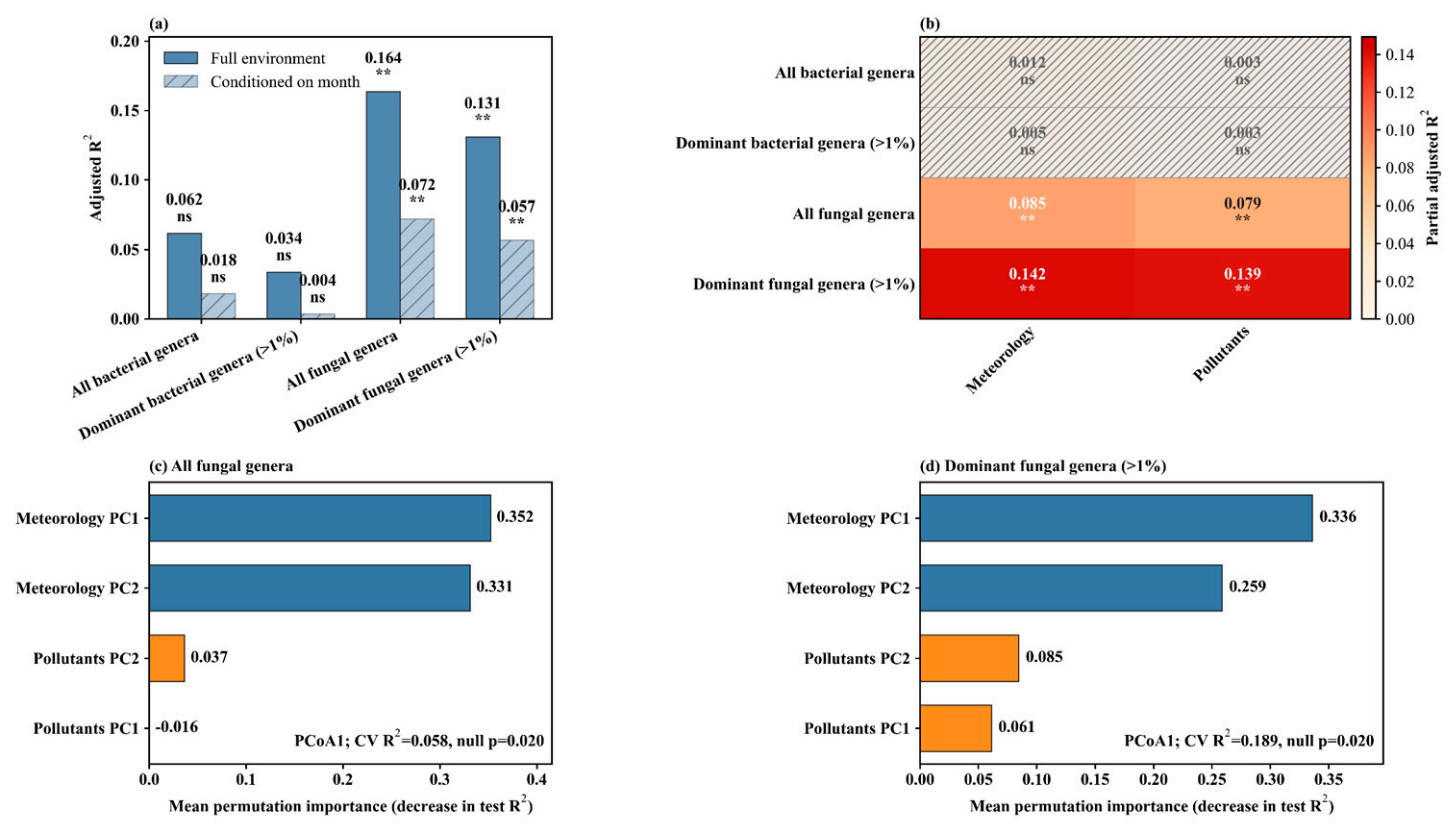
Environmental explanatory power for bacterial and fungal communities. (**a**) db-RDA of Bray–Curtis dissimilarities from Hellinger-transformed abundances, using PCA axes for meteorology and gaseous pollutants. Bars show adjusted *R^2^* for full and month-conditioned models. (**b**) Partial adjusted *R^2^* by module. Hatched bars denote unstable bacterial models, asterisks indicate BH-FDR *q* < 0.05. Permutation importance of environmental PCA axes in ABT models of fungal PCoA1 for all (**c**) and dominant genera (**d**); larger test set *R^2^* declines indicate greater importance. “All bacterial genera” and “all fungal genera” refer to all genera retained after taxonomic preprocessing. “Dominant bacterial genera (>1%)” and “dominant fungal genera (>1%)” were defined as genera with a mean relative abundance > 1% across the 68 samples included in the background environmental analysis. In panels (**a**,**b**), ** indicates *q* < 0.01, and ns indicates *q* ≥ 0.05.

Bacterial and fungal communities exhibited distinct responses to environmental gradients (Figure 3a). The explanatory power of environmental models for bacteria was limited (adjusted *R^2^* = 0.062 for all genera; 0.034 for dominant genera), and conditional tests controlling for temporal effects (date and month) lacked statistical significance. Specifically, bacterial ABT results were excluded because their cross-validated *R^2^* values fell below the lower bound of the null model’s 95% CI, indicating predictive performance did not exceed random expectation. Therefore, robust environmental associations could not be established for bacteria, as further evidenced by their negligible partial *R^2^* values. Conversely, fungal communities were more robustly coupled with environmental factors (month-adjusted *R^2^* = 0.072 and 0.057 for all and dominant genera, respectively). Partial RDA demonstrated significant independent contributions from meteorology, gaseous pollutants to all-genus fungal variation (partial *R^2^* = 0.085 and 0.079, respectively; *p* < 0.05), with even stronger signals among dominant genera (0.142, and 0.139). Consistent with previous findings that fungal aerosolization depends heavily on ambient meteorological and surface conditions [1,2,59], our analysis highlights meteorology and pollution as the primary drivers of fungal composition at this site.

Bacterial communities exhibited no robust environmental associations at either the overall or module-specific levels. However, this lack of statistical correlation should not be interpreted as an inherent insensitivity to environmental drivers. Rather, it likely stems from the high heterogeneity of bacterial emission sources, the influence of unmeasured variables, or complex atmospheric processing. Indeed, the previous literature indicates that environmental drivers of airborne bacteria are highly context-dependent. For instance, while meteorology was identified as the primary driver of bacterial community variation in Beijing [60], air pollutants and PM_2.5_ chemistry accounted for the greatest variance in the broader Beijing–Tianjin–Hebei region [61]. Such discrepancies across studies underscore the profound influence of spatiotemporal factors (e.g., location, season, and ambient pollution levels) and methodological differences (e.g., targeted particle size fractions, sampling duration, and analytical techniques) on the observed bioaerosol dynamics.

#### 3.2.2. Environmental Predictors of Fungal Communities

ABT models also identified meteorological gradients as the strongest predictors of fungal PCoA1 scores (Figure 3c,d). PCoA1 explained 24.6% of all-genus and 33.3% of dominant-genus fungal variation. As detailed in Table 1, Meteorology PC1 represented stronger boundary layer development and radiation with drier conditions. Meteorology PC2 represented higher pressure, wind speed, and boundary layer height, together with shifts in temperature and humidity. Permutation importance values for Meteorology PC1 and PC2 were 0.352 and 0.331 for all genera, and 0.336 and 0.259 for dominant genera, respectively. These values substantially exceeded gaseous pollutants. Thus, the main fungal gradient was most predictably associated with meteorological conditions, while gaseous pollutants contributed comparatively little out-of-sample importance.

Meteorology PC1 integrates boundary layer development, solar radiation, and atmospheric dryness, thereby encapsulating multiple physical processes such as vertical mixing, regional transport, and radiation stress. Meteorology PC2 is thought to capture variations in spore release, dispersion, and deposition governed by wind speed and barometric pressure. Notably, neither axis represents the effect of a single isolated factor, but rather a combined meteorological gradient. Meteorological associations have been reported in built environments [62,63] and outdoor urban air [20,64]. Our findings build upon these observations, revealing that integrated meteorological gradients exhibit the most robust predictive power for fungal PCoA1 within this urban environment. Similarly, Geng et al. [15] observed less pronounced meteorological covariation when diel temperature and humidity gradients were limited, suggesting that the detectability of these associations strongly depends on the amplitude of the sampled environmental fluctuations.

The gaseous pollutant module showed significant partial covariation with fungal community composition, but its ABT importance was substantially lower than that of meteorology. O_3_, NO_2_, SO_2_, and CO reflect photochemical and combustion-related processes, while also covarying with wind, humidity, boundary layer development, and atmospheric transport. Such coupling between meteorological conditions, pollution, local emissions, and transport has also been reported for airborne microbial communities [50]. The partial RDA signal should therefore be interpreted primarily as fungal community covariation with a coupled atmospheric pollution regime.

### 3.3. Assessment of Relative Exposure-Relevant Proportion

#### 3.3.1. Composition of RERP

We focused on seven fungal genera with potential exposure relevance, such as *Cladosporium*, *Alternaria*, *Aspergillus*, *Talaromyces*, *Fusarium*, *Curvularia*, and *Penicillium*. These genera have been discussed in relation to fungal allergenicity, opportunistic pathogenicity, or urban airborne occurrence [6,7,65]. They also had high occurrence, substantial relative abundance, or clear temporal and environmental signals in this dataset. It is important to reiterate that the RERP is a compositional index derived from relative abundance data. It reflects the relative contribution of selected genera, not absolute spore concentration, deposition flux, or inhaled dose. The RERP comprised a large fraction of fungal sequence reads and differed by diel period (Figure S2a). Mean values were 46.9% for cold–day, 31.3% for cold–night, 52.0% for warm–day, and 18.6% for warm–night. The RERP was significantly higher during the day, whereas the overall cold–warm contrast was not significant. Thus, diel variation was more evident than the broad seasonal contrast.

*Cladosporium* was the largest contributor to the RERP in all groups, whereas *Alternaria* contributed more during the warm season (Figure S2b). *Aspergillus*, *Talaromyces*, and *Fusarium* also contributed in some groups, showing that the RERP was a composite rather than a single-genus measure. Long-term spore monitoring has identified *Cladosporium* and *Alternaria* as common airborne allergenic genera with pronounced daytime peaks [66,67]. Higher daytime RERP is consistent with a larger relative contribution from dry-discharged spores [67]. Stronger radiation, lower humidity, and greater turbulence may contribute to this pattern. Lower nighttime RERP does not indicate lower fungal concentration or exposure. Instead, it coincided with the relative enrichment of basidiomycetes and wood decay-associated fungi described in Section 3.1.2. The convergence of higher nocturnal humidity, attenuated atmospheric turbulence, and a compressed boundary layer creates conducive conditions for wet-discharged spores, ultimately modulating the overall community structure [56,58].

#### 3.3.2. Environmental Associations of RERP and Selected Genera

**Figure 4:**
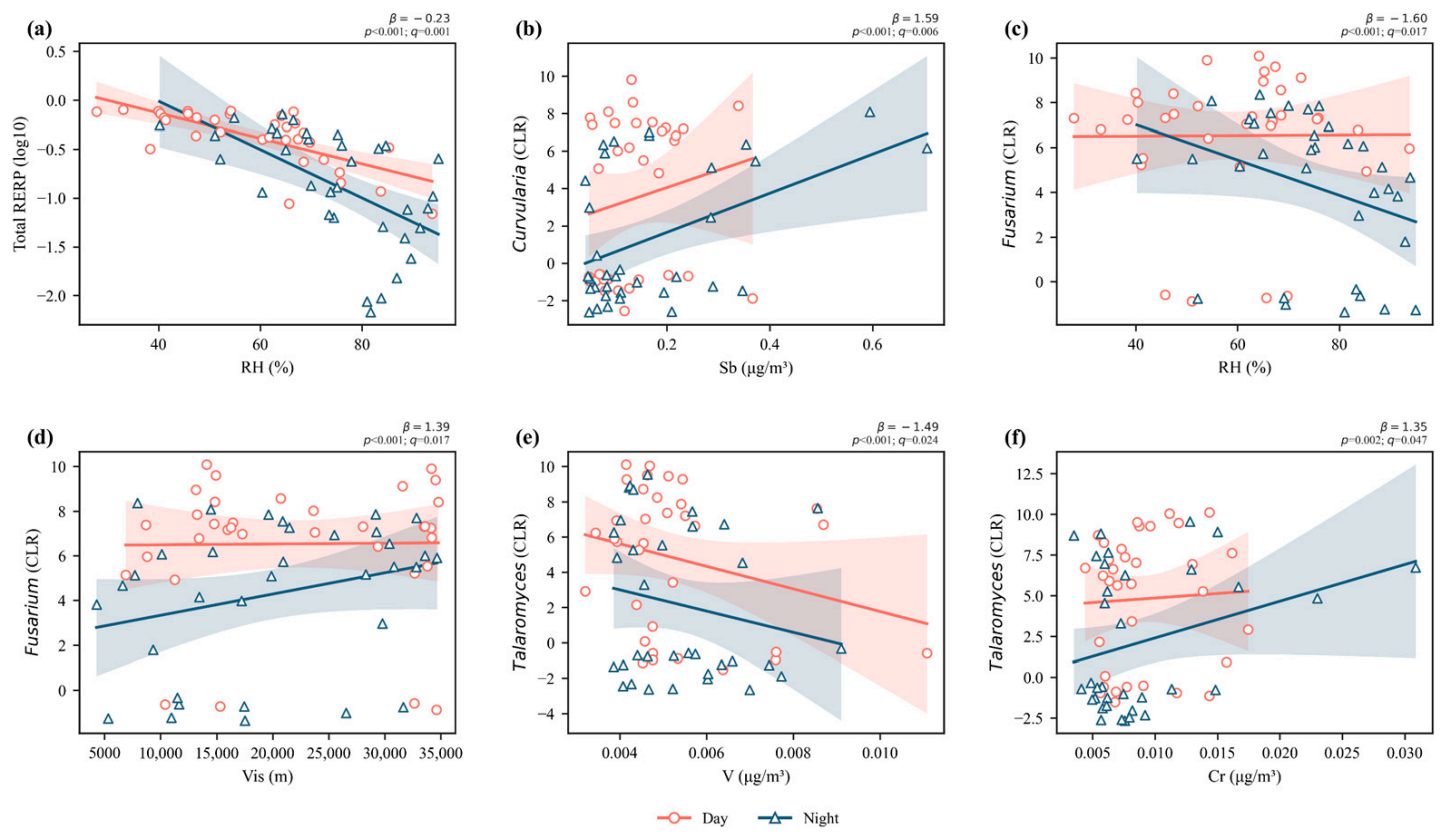
Linear associations of the Relative Exposure-Relevant Proportion (RERP) and key fungal genera with environmental variables. (**a**) Total RERP vs. relative humidity (RH), (**b**) *Curvularia* vs. Sb, (**c**) *Fusarium* vs. RH, (**d**) *Fusarium* vs. visibility (Vis), (**e**) *Talaromyces* vs. V, and (**f**) *Talaromyces* vs. Cr. Circles and triangles denote day and night samples. Lines and shading show diel-specific fits and 95% CIs. Upper-right *β*, *p*, and *q* values are model outputs; only associations with *q* < 0.05 are shown.

We applied linear mixed-effects models to investigate how meteorological parameters and gaseous pollutants relate to the RERP and targeted fungal genera, while examining the potential covariation in PM_2.5_ chemical constituents as an exploratory supplement. After adjustment for diel period and month, the total RERP was negatively associated with relative humidity (*β* = −0.23, *q* = 0.001; Figure 4a). This conditional association is consistent with reports that *Cladosporium* and *Alternaria* are relatively abundant under drier conditions [66,68]. Dry conditions may favor the detachment of dry-discharged spores, whereas high humidity may increase the relative contribution of wet-discharged fungi [56]. Due to the compositional structure of the RERP and the covariation in environmental parameters, this coefficient precludes the establishment of a direct causal link with humidity.

Associations varied among the selected genera (Table 2; Figure 4). A significant positive association was observed between *Curvularia* and Sb (*β* = 1.59, *q* = 0.006), suggesting that its abundance covaries with road dust or traffic-related particles, as Sb is a recognized indicator of non-exhaust traffic emissions like brake wear [3]. However, the association is conditional and does not establish a direct traffic source or causal effect on *Curvularia*. *Talaromyces* was negatively associated with V (*β* = −1.49, *q* = 0.024) and positively associated with Cr (*β* = 1.35, *q* = 0.047). Although V is commonly used as a tracer of heavy-fuel-oil and ship emissions, the contrasting relationships with V and Cr suggest that these metals are better interpreted here as markers of the contemporaneous chemical environment rather than as direct indicators of fungal sources.

*Fusarium* was negatively associated with relative humidity (*β* = −1.60, *q* = 0.017) and positively associated with visibility (*β* = 1.09, *q* < 0.01). These relationships are consistent with greater aerosolization of plant- or soil-associated spores under drier, better-dispersed conditions [2,56]. Overall, the total RERP and individual genera showed distinct conditional associations, but these single-predictor models do not demonstrate independent environmental effects.

### 3.4. Community Changes During a Strong Cold-Air Event

#### 3.4.1. Air Mass and Physicochemical Transitions

**Figure 5:**
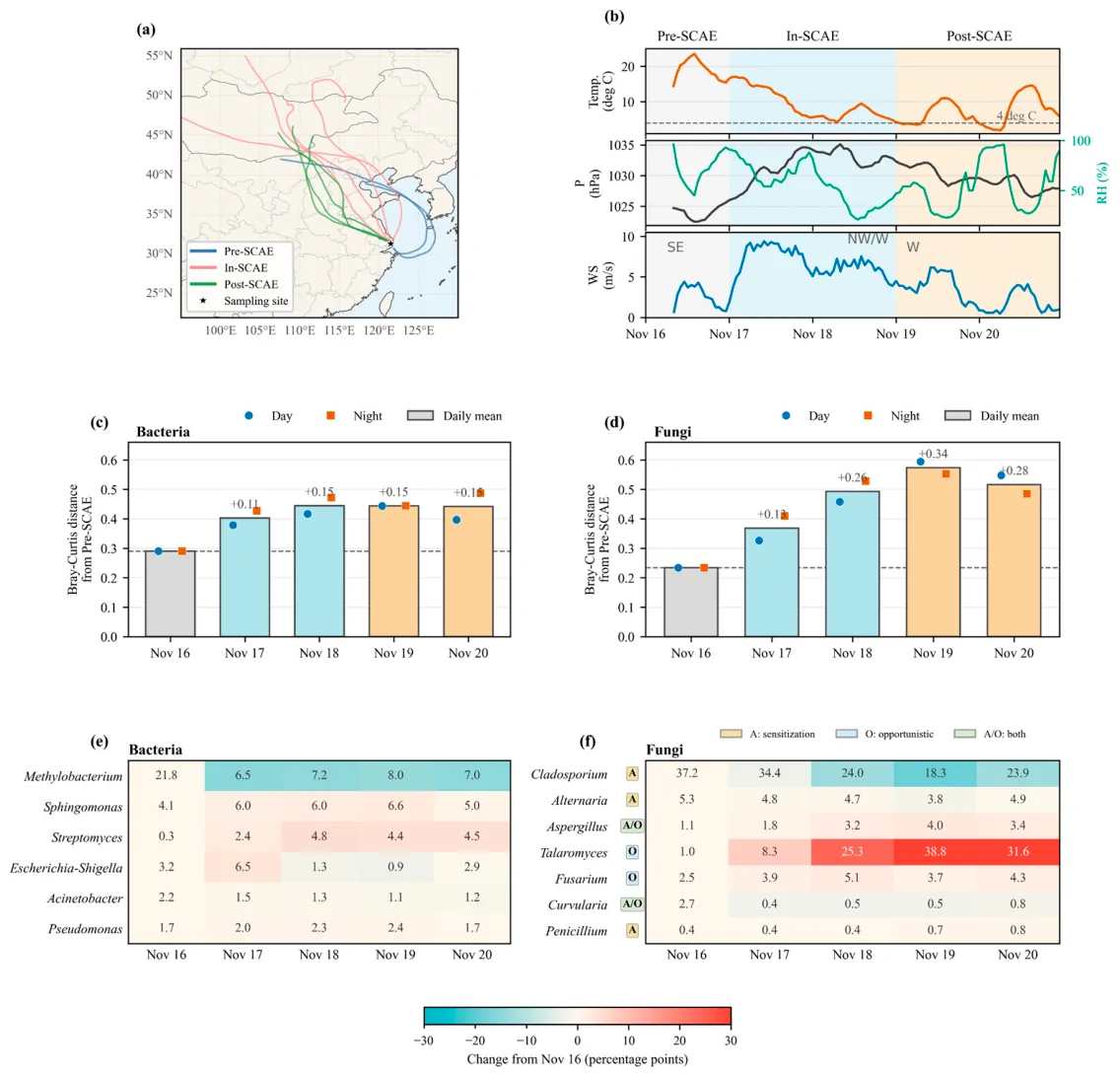
Air mass, physicochemical, and microbial changes during SCAE (strong cold-air event; Pre/In/Post: before/during/after). (**a**) 72 h back trajectories, 16–20 November 2025; colors mark stages and the star marks the site. (**b**) The orange, dark-gray, green, and blue lines represent temperature (Temp), atmospheric pressure (P), relative humidity (RH), and wind speed (WS), respectively; background colors indicate the three event stages, the gray dashed line marks the 4 °C minimum-temperature reference threshold, and in-panel labels indicate prevailing wind directions (**c**,**d**). Bray–Curtis distances from the 16 November day/night centroid; bars are daily means and symbols denote day/night. (**e**,**f**) Daily mean abundances; values are percentages and colors show changes from 16 November. A, allergenic/sensitizing, O, genera containing opportunistic pathogens.

We used one strong cold-air event to describe short-term community changes outside the background sampling conditions. By evaluating hourly variations in atmospheric pressure, wind speed, and wind direction against the GB/T 20484-2017 criteria [69], the meteorological conditions observed between 16 and 20 November 2025 were officially classified as a strong cold-air event (SCAE). The temperature fell by 8.8 °C over 24 h and 10.2 °C over 48 h. The daily minimum fell below 4 °C late in the event. Air mass back trajectories shifted from slow local or offshore transport prior to frontal passage, and to northern and northwestern continental transport during and following the cold-air intrusion (Figure 5a). The daily minimum temperature decreased from 14.4 °C to 1.9 °C. The mean sea-level pressure reached 1033.2 hPa, and the mean wind speed reached 7.65 m/s on 17 November. Prevailing winds shifted from southeast to northwest (Figure 5b). The PM_2.5_ major chemical composition also changed (Table 3), with increases in NO_3_^−^ (3.45-fold), secondary inorganic aerosol (SNA; 2.28-fold), and Cu (2.46-fold). To provide a more complete characterization of the physicochemical evolution during the strong cold-air event, daily variations in temperature, relative humidity, atmospheric pressure, wind speed, prevailing wind direction, gaseous pollutants, and PM_2.5_ chemical components are summarized in Table S7. The frontal passage was marked by rapid cooling, increasing pressure, strengthened winds, and a transition in prevailing airflow from southeasterly to northwesterly–westerly directions. In contrast, several particulate chemical components, including secondary inorganic aerosol (SNA), OC, EC, and trace metals, increased primarily during the later stage of the event, indicating that the meteorological transition and particulate chemical response were not fully synchronous. These concurrent changes provide the environmental context for the microbial observations. Previous work has linked airborne community composition with transport and circulation [33,70]. Other studies reported covariation with season and particle chemistry [2,9,11].

#### 3.4.2. Contrasting Shift Dynamics of Bacterial and Fungal Communities

Given the restricted sample size associated with this single event, we refrained from conducting statistical tests among stages to avoid underpowered inferences. Instead, we calculated each sample’s Bray–Curtis distance from the pre-event community centroid. The mean bacterial distance increased from 0.29 on 16 November to 0.40 on 17 November and remained at 0.44–0.45 from 18 to 20 November (Figure 5c). The initial increase coincided with cooling, rising pressure, strong winds, and air mass replacement. During this phase, the SNA levels (12.70 μg/m^3^) were consistent with the pre-event baseline values (12.79 μg/m^3^; Table 3). This timing implies a role for transport and mixing, though the study design cannot decouple these mechanisms. Notably, wind shifts and regional transport are known to induce rapid shifts in airborne bacterial composition [70,71].

The dissimilarity of fungal communities exhibited a gradual upward trend, increasing from an initial value of 0.23 on 16 November to a maximum of 0.57 on 19 November, followed by a decrease to 0.52 on 20 November (Figure 5d). The larger late-event offsets coincided with a lower wind speed, a shallower boundary layer, and higher NO_3_^−^, NH_4_^+^, SNA, OC, EC, and several metals in PM_2.5_ (Table 3). These chemical changes are interpreted only as part of the evolving atmospheric background. Fungal communities have been associated with aerosol organic components, release conditions, and micrometeorology [55]. The observed pattern may therefore reflect several concurrent changes in release, transport, retention, and deposition. Different event types can produce different microbial responses [72,73]. Bacterial composition responded acutely to the cold-air intrusion, contrasting with the more gradual temporal trajectory of fungal communities. Given the observational nature of this single-event analysis, confirming this differential response necessitates replication across subsequent cold-air events.

#### 3.4.3. Changes in Dominant Genera

Both bacterial and fungal genera changed during the event, but their temporal patterns differed. Bacterial changes appeared early and included a decline in the plant- or surface-associated genus *Methylobacterium* [74]. The soil- or dust-associated genus *Streptomyces* increased later [75]. These patterns are consistent with changing air masses and source contributions but do not identify a specific source. Fungal changes involved reciprocal shifts among dominant genera, particularly *Cladosporium* and *Talaromyces*. This sequence may reflect combined changes in fungal release, transport, and retention during the weather transition.

The internal composition of the RERP also changed during the event (Figure 5f). The RERP increased from approximately 47% under the background conditions to nearly 69%, while its dominant contributors shifted. *Cladosporium*, a commonly reported allergenic genus, contributed strongly under background conditions [41,42,66]. During the later event stage, *Talaromyces* became the largest contributor. Some *Talaromyces* species are opportunistic pathogens, but genus-level sequencing cannot identify *T. marneffei* or establish pathogenic potential in the sampled material. The event therefore coincided with a shift in the relative composition of the RERP indicator. It does not demonstrate an increase in absolute fungal exposure or health risk.

## 4. Conclusions

Airborne bacterial and fungal communities showed distinct patterns of seasonal, diel, and event scales. Fungal composition exhibited more pronounced variations across seasonal and diurnal scales, demonstrating stronger covariation with meteorological gradients. Gaseous pollutants were also associated with fungal composition, but their contributions overlapped with meteorological and particulate backgrounds. The RERP values were elevated during the daytime and exhibited a negative association with relative humidity, even after controlling for month and diel period. During one strong cold-air event, bacterial composition shifted rapidly, whereas fungal composition changed more gradually. These observations describe covariation at one site and provide hypotheses for longer, multi-site studies of urban bioaerosols.

While this study provides comprehensive observational insights, several caveats highlight avenues for future research. First, the differential responses to the cold-air intrusion are based on a single well-characterized event; establishing the generalizability of these dynamics necessitates replication across multiple weather systems. Second, the limited explanatory power for bacterial variation suggests that their community assembly is likely governed by highly localized source emissions and complex turbulent transport not fully captured by macroscopic environmental modules. Future campaigns should incorporate absolute quantification (e.g., qPCR), specific source tracing, and finer-scale turbulence measurements. Third, a data resolution mismatch exists between our microbial (pooled size fractions) and PM_2.5_ chemical measurements. Consequently, our chemical data are used only to represent the ambient fine-particle background, not as a direct proxy for the microbe-carrying particles. Size-matched simultaneous sampling will be required in future studies to explore such particle-specific links. Finally, because amplicon sequencing provides only genus-level relative abundances, the RERP should be viewed as an ecological indicator rather than a direct metric of absolute concentration, biological viability, or inhalation risk. Coupling metagenomic and metatranscriptomic approaches with coordinated, multi-site continuous monitoring will be essential to translate these compositional patterns into quantitative health impact assessments.

## Figures and Tables

**Table 1:** Main loadings and ecological interpretations of PCA axes for environmental modules. Abbreviations: BLH, boundary layer height; RH, relative humidity; Temp, temperature; WS, wind speed; SSRDC, surface solar radiation downwards under clear-sky conditions.

Module	PC Axis	Explained Variance	Main Positive Loadings	Main Negative Loadings	Ecological Interpretation
Meteorology	PC1	32.6%	BLH, SSRDC, Vis, Temp	RH, P	boundary layer/radiation dryness gradient
Meteorology	PC2	20.9%	P, WS, BLH	Temp, RH	pressure cool/moistness gradient
Pollutants	PC1	44.1%	CO, NO_2_, SO_2_	O_3_	combustion/traffic pollution gradient
Pollutants	PC2	28.5%	CO, O_3_, SO_2_	NO_2_	photochemical/secondary pollution gradient

**Table 2:** Interaction terms between environmental predictors and diel period (night vs. day) from LMMs for core fungal genera. βint quantifies the night–day slope difference; positive = steeper (or less negative) slope at night. Statistical details (*β*int, 95% CI, P, BH-adjusted *q*, *β*Day, *β*Night) are summarized in the table. Models control for month (fixed) and sampling date (random intercept).

Genus	Variable	*β*int	95% CI	*p*	*q*	*β*Day	*β*Night
*Coprinellus*	Temp	0.662	[0.404, 0.920]	<0.001	<0.001	0.001	0.662
*Cladosporium*	Temp	−0.325	[−0.538, −0.112]	0.003	0.081	0.477	0.152
*Coprinellus*	P	−0.539	[−0.823, −0.254]	<0.001	0.017	−0.136	−0.675
*Cladosporium*	NO_2_	0.449	[0.189, 0.709]	<0.001	0.028	−0.465	−0.016
*Cladosporium*	Fe	0.451	[0.193, 0.709]	<0.001	0.027	−0.125	0.326
*Irpex*	Cd	0.318	[0.189, 0.448]	<0.001	<0.001	−0.342	−0.023
*Trametes*	Sb	0.708	[0.378, 1.038]	<0.001	0.002	−0.643	0.064
*Trametes*	Cd	0.468	[0.281, 0.655]	<0.001	<0.001	−0.373	0.095

**Table 3:** Changes in meteorological conditions and chemical components across the strong cold-air event. Values are summarized for the pre-SCAE, in-SCAE, and post-SCAE periods, with the post/pre ratio indicating the magnitude of change relative to the pre-event baseline. Units are shown in the variable names where applicable. SCAE, strong cold-air event.

Variable	Pre-SCAE (16 November)	In-SCAE (17–18 November)	Post-SCAE (19–20 November)	Post/Pre Ratio
SO_4_^2−^ (µg/m^3^)	3.17	2.37	3.22	1.02
NO_3_^−^ (µg/m^3^)	5.18	5.94	17.89	3.45
NH_4_+ (µg/m^3^)	4.44	4.39	8.03	1.81
SNA (µg/m^3^)	12.79	12.70	29.15	2.28
OC (µg/m^3^)	3.03	2.74	5.24	1.73
EC (µg/m^3^)	1.23	0.73	2.33	1.90
Fe (µg/m^3^)	0.20	0.22	0.31	1.54
Sb (µg/m^3^)	0.10	0.12	0.19	1.93
Cu (µg/m^3^)	0.07	0.11	0.18	2.46

## Data Availability

The raw data supporting the conclusions of this article will be made available by the authors on request.

## Supplementary Materials

The following supporting information can be downloaded at: https://www.mdpi.com/article/10.3390/microorganisms14091975/s1. Text S1. Materials and Methods. Text S1.1. Quality assurance and quality control (QA/QC). Text S1.2. DNA extraction, amplification, and sequencing. Figure S1. Monthly and diel variation in environmental conditions. (a) Principal component analysis (PCA) of standardized environmental variables. Colors indicate sampling months, and circles and triangles denote day and night samples, respectively. Shaded ellipses summarize the distributions of the two diel groups. ANOSIM results for month and diel period are shown in the upper-left corner. (b) Standardized distributions of environmental variables showing significant differences between day and night after BH-FDR correction, including Ti, SO_4_^2−^, temperature (Temp), relative humidity (RH), wind speed (WS), boundary layer height (BLH), clear-sky surface downward solar radiation (SSRDC), O_3_, NO_2_, and SO_2_. Variables were analyzed as raw or log1p-transformed values and subsequently z-standardized. Boxes indicate interquartile ranges, center lines indicate medians, and whiskers show data dispersion. Asterisks denote BH-adjusted significance levels: *q* < 0.05 *, *q* < 0.01 **, and *q* < 0.001 ***. Only variables with *q* < 0.05 are shown in panel (b); Figure S2. Variation in the composition of the Relative Exposure-Relevant Proportion (RERP). (a) RERP as a percentage of the fungal community across the four season-by-diel groups. Boxes represent the interquartile range, center lines indicate the median, whiskers extend from the 10th to the 90th percentile, and points represent individual samples. (b) Contributions of the target genera to total RERP in the four season-by-diel groups. The area of each sector represents the proportion of the cumulative relative abundance of the corresponding genus in the total RERP within each group. Only genera contributing at least 5% are labeled; Table S1. Specific sampling dates and retained daytime and nighttime sample numbers for each sampling campaign; Table S2. Particle size range and classification parameters of the sampler. Aperture size, airflow velocity, nominal collection range are listed for each sampler stage. Note that samples were pooled across stages and were not used for size-resolved community analyses; Table S3. Variable composition, Maximum initial variance inflation factor (max VIF), screening strategy, and final predictors used in RDA for each environmental module. For the Meteorology module, variables include temperature (Temp), relative humidity (RH), wind speed (WS), air pressure (P), visibility (Vis), boundary layer height (BLH), surface solar radiation downward clear-sky (SSRDC), and the sine and cosine transformations of wind direction (WD_sin, WD_cos). For the Gaseous Pollutants module, variables include O_3_, NO_2_, SO_2_, CO; Table S4. Seasonal and diel patterns of bacterial and fungal α-diversity metrics under background sampling conditions; Table S5. Statistical tests for monthly and day–night differences in alpha- and beta-diversity indices; Table S6. Monthly mean values of meteorological parameters and chemical components during the study period. Values summarize matched sampling months from November 2024 to November 2025. Units are shown in the variable names where applicable. The table provides the environmental background used to interpret seasonal community variation and downstream genus-environment association analyses; Table S7. Daily meteorological conditions and PM_2.5_ chemical composition during the strong cold-air event in Shanghai, 16–20 November 2025.
