# Supplementary material for: Temporal Dynamics of Airborne Bacterial and Fungal Communities in Megacity Shanghai: Diel Asynchrony, Environmental Drivers, and Cold Front Perturbations
Source: Microorganisms. 2026 Sep 7;14(9):1975. doi: 10.3390/microorganisms14091975 (PMC13609338; doi:10.3390/microorganisms14091975)
Supplement: Supplementary file 1 [file microorganisms-14-01975-s001.zip › microorganisms-4511786-supplementary.pdf]

Supplementary data

Contents of the file

Number of Pages: 14

Number of figures: 2

Number of tables: 7

Text S1 Materials and Methods

Text S1.1 Quality assurance and quality control (QA/QC)

To ensure the precision and reproducibility of the data, a comprehensive quality assurance and quality control (QA/QC) framework was strictly implemented.

(i) Instrumental Calibration and Flow Stability: The volumetric flow rate of the ZR-2000 sampler was dynamically calibrated using a metrologically certified soap-film flowmeter. Calibrations were performed prior to each sampling batch and at bi-weekly intervals to maintain a flow rate tolerance within ±5%. During active collection, the pump performance was routinely monitored to mitigate potential concentration biases arising from flow fluctuations.

(ii) Decontamination and Aseptic Maintenance: Rigorous sterilization of the sampling apparatus was conducted to prevent cross-contamination. The sampler inlet was disinfected with absolute ethanol after each session. Furthermore, internal sampling lines and gaskets underwent a tri-weekly cleaning regimen involving absolute ethanol irrigation followed by triple ultrasonic cleaning, ensuring they were completely desiccated before reuse.

(iii) Procedural Controls and Background Monitoring: To scrutinize potential exogenous microbial interference, a dual-control strategy was employed. Negative controls (consisting of molecular-grade water) were integrated into every stage of the downstream processing, from DNA extraction through PCR amplification. Simultaneously, field blanks were deployed during each sampling event to evaluate the efficacy of sterilization and the influence of the environmental background. These field blanks were mounted on the sampler for the same duration as the active samples without airflow, and subsequently subjected to identical analytical workflows, including DNA extraction and PCR amplification of the ITS region. However, field blanks were not subjected

to high-throughput sequencing as no detectable target amplicons were observed on agarose gels, this result reduced the likelihood of major contamination, but low-level reagent contamination cannot be completely excluded. This rigorous benchmarking substantiated the integrity of the collected samples and confirmed that any detected taxa were representative of the atmospheric fungal communities rather than laboratory or transport-acquired artifacts.

Prior to bioinformatic processing, rigorous quality control (QC) was performed on negative controls (extraction and PCR blanks). The absence of detectable DNA amplicons in these control samples confirmed the efficacy of contamination mitigation during sampling and extraction, ensuring the integrity of the downstream analyses. For field blanks, the absence of visible amplicons precluded them from sequencing, and they were thus excluded from downstream community analyses.

Text S1.2 DNA extraction, amplification, and sequencing

The extraction of total genomic DNA was carried out using the MJ Water DNA Extraction Kit (Shanghai Meiji Biomedical Technology Co., Ltd., China) in accordance with the manufacturer's instructions. The extracted DNA was stored at -80 °C until further use. For bacterial 16S rRNA gene amplification, the V3-V4 hypervariable regions were targeted using primers 338F (5'-ACTCCTACGGGAGGCAGCAG-3') and 806R (5'-GGACTACHVGGGTWTCTAAT-3'). For fungal ITS region amplification, the ITS1 region was amplified using primers ITS1F (5'-CTTGGTCATTTAGAGGAAGTAA-3') and ITS2/ITS2R (5'-GCTGCGTTCTTCATCGATGC-3'). Both PCR reactions were performed in a total volume of 20 μL containing 4 μL of 5x TransStart FastPfu buffer, 2 μL of 2.5 mM dNTPs, 0.8 μL of each primer (5 μM), 0.4 μL of TransStart FastPfu DNA polymerase, and 10 ng of template DNA. Amplification was carried out on an ABI GeneAmp 9700 thermocycler under the following conditions: initial denaturation at 95 °C for 3 min; followed by 27 cycles for bacteria or 35 cycles for fungi of denaturation at 95 °C for 30 s, annealing at 55 °C for 30 s, and extension at 72 °C for 30 s; with a final extension at 72 °C for 10 min and holding at 4 °C.

PCR products were checked by 2% agarose gel electrophoresis, and target bands were purified using a DNA gel recovery kit (PCR Clean-Up Kit, Yuhua, China). The purified products were quantified using Qubit 4.0 (Thermo Fisher Scientific, USA). Sequencing libraries were constructed using the NEXTFLEX Rapid DNA-Seq Kit, including adapter ligation, magnetic bead-based size selection to remove adapter dimers, PCR enrichment of library templates, and final magnetic bead purification. The libraries were sequenced on the Illumina NextSeq 2000 platform at Shanghai Meiji Biomedical Technology Co., Ltd. A total of 78 samples successfully passed PCR amplification for

both bacterial and fungal amplicons.

For bioinformatics processing, raw paired-end reads were quality-filtered using FASTP version 0.23.4. Reads were trimmed from the 3' end using a 10-bp sliding window and cut when the average quality score dropped below 20; reads shorter than 50 bp after trimming or containing ambiguous N bases were discarded. Paired-end reads were merged using FLASH version 1.2.11, with a minimum overlap of 10 bp and a maximum mismatch ratio of 0.2 in the overlapping region. Sequences were demultiplexed and assigned to samples based on barcodes and primers, allowing zero mismatches for barcodes and up to two mismatches for primers. Operational taxonomic units (OTUs) were clustered at 97% sequence similarity using UPARSE version 11, and chimeric sequences were removed. For bacterial sequences, reads annotated as chloroplast or mitochondrial sequences were excluded. Taxonomic assignment was performed using the RDP Classifier version 2.11 against the SILVA database version 138.2, with a confidence threshold of 70% for bacteria, and against the UNITE database version 8.2, with a confidence threshold of 95% for fungi. All samples were rarefied to 20,000 sequences per sample to standardize sequencing depth. Data analysis was performed using the Majorbio Cloud (www.majorbio.com).

Figure S1. Monthly and diel variation in environmental conditions. (a) Principal component analysis (PCA) of standardized environmental variables. Colors indicate sampling months, and circles and triangles denote day and night samples, respectively. Shaded ellipses summarize the distributions of the two diel groups. ANOSIM results for month and diel period are shown in the upper-left corner. (b) Standardized distributions of environmental variables showing significant differences between day and night after BH-FDR correction, including Ti, SO_4^{2-}, temperature (Temp), relative humidity (RH), wind speed (WS), boundary layer height (BLH), clear-sky surface downward solar radiation (SSRDC), O_3, NO_2, and SO_2. Variables were analyzed as raw or log1p-transformed values and subsequently z-standardized. Boxes indicate interquartile ranges, center lines indicate medians, and whiskers show data dispersion. Asterisks denote BH-adjusted significance levels: $q < 0.05$ *, $q < 0.01$ **, and $q < 0.001$ ***. Only variables with $q < 0.05$ are shown in panel (b).

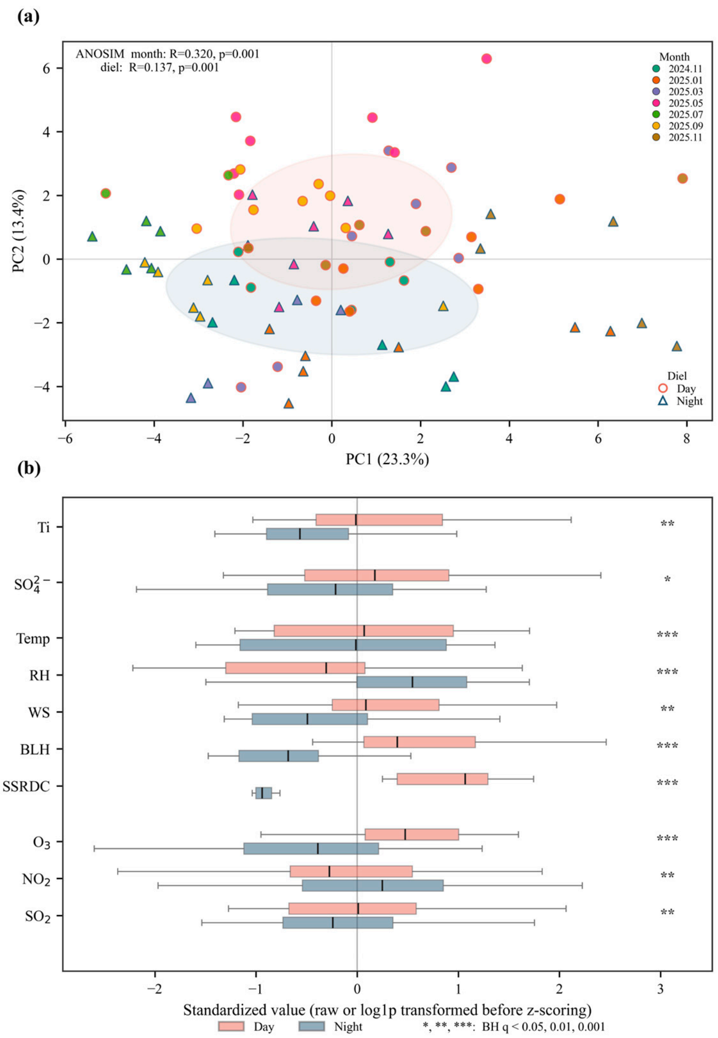

Figure S2. Variation in the composition of the Relative Exposure-Relevant Proportion (RERP). (a) RERP as a percentage of the fungal community across the four season-by-diel groups. Boxes represent the interquartile range, center lines indicate the median, whiskers extend from the 10th to the 90th percentile, and points represent individual samples. (b) Contributions of the target genera to total RERP in the four season-by-diel groups. The area of each sector represents the proportion of the cumulative relative abundance of the corresponding genus in the total RERP within each group. Only genera contributing at least 5% are labeled.

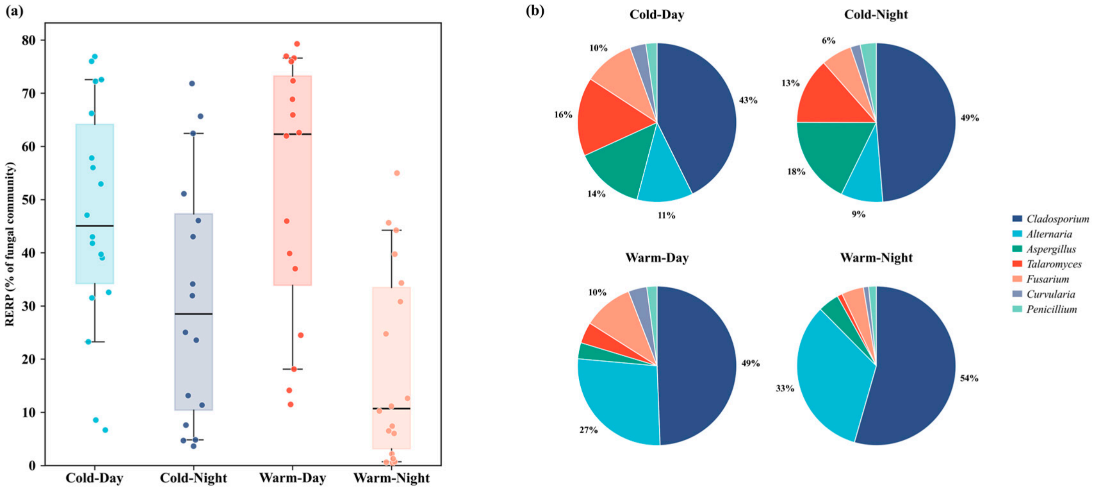

Table S1 Specific sampling dates and retained daytime and nighttime sample numbers for each sampling campaign.

**Sampling campaign**	**Specific sampling dates**	**Samples (n)**	**Analysis set**
November 2024	2024-11-02 (N); 03 (D/N); 04 (D/N); 05 (D/N); 06 (D); 08 (D/N)	10	Background analysis
January 2025	2025-01-01 (D/N); 02 (D/N); 03 (D/N); 04 (N); 05 (D/N); 06 (D/N); 07 (D/N)	13	Background analysis
March 2025	2025-03-08 (D); 09 (D/N); 10 (D); 11 (D/N); 16 (D/N); 17 (D); 18 (D/N)	11	Background analysis
May 2025	2025-05-11 (D); 12 (N); 13 (D/N); 14 (D/N); 25 (D/N); 26 (D/N); 27 (D/N); 30 (N); 31 (D)	14	Background analysis
July 2025	2025-07-05 (N); 06 (N); 22 (D/N); 23 (N); 24 (D/N)	7	Background analysis
September 2025	2025-09-04 (D/N); 05 (D/N); 06 (D/N); 07 (D/N); 08 (D); 09 (D/N); 10 (D/N)	13	Background analysis
November 2025	2025-11-16 (D/N); 17 (D/N); 18 (D/N); 19 (D/N); 20 (D/N)	10	Strong cold-air event

Total	—	78	68 background + 10 event

Table S2. Particle size range and classification parameters of the sampler. Aperture size, airflow velocity, nominal collection range are listed for each sampler stage. Note that samples were pooled across stages and were not used for size-resolved community analyses.

**Aperture (mm)**	**Air Flow Velocity (m/s)**	**Collection Range (μm)**
1.18	1.1	> 7.0
0.91	1.8	4.7 ~ 7.0
0.71	3.0	3.3 ~ 4.7
0.53	5.3	2.1 ~ 3.3
0.34	13.0	1.1 ~ 2.1
0.25	24.0	0.65 ~ 1.1

Table S3. Variable composition, Maximum initial variance inflation factor (max VIF), screening strategy, and final predictors used in RDA for each environmental module. For the Meteorology module, variables include temperature (Temp), relative humidity (RH), wind speed (WS), air pressure (P), visibility (Vis), boundary layer height (BLH), surface solar radiation downward clear-sky (SSRDC), and the sine and cosine transformations of wind direction (WD_sin, WD_cos). For the Gaseous Pollutants module, variables include O_3, NO_2, SO_2, CO.

<table>
<tr><th>Index</th><th>Meteorology</th><th>Gaseous Pollutants</th></tr>
<tr><td>Variables</td><td>Temp, RH, WS, P, Vis, BLH, SSRDC, WD_sin, WD_cos</td><td>O_3, NO_2, SO_2, CO</td></tr>
<tr><td>max VIF</td><td>4.838</td><td>1.751</td></tr>
<tr><td>Screening strategy</td><td colspan="2">PCA axis</td></tr>
<tr><td>RDA input variables</td><td colspan="2">PC1, PC2</td></tr>
<tr><td>Final max VIF</td><td colspan="2">1.000</td></tr>
<tr><td>Explained variance</td><td>PC1 32.6%, PC2 20.9%</td><td>PC1 44.1%, PC2 28.5%</td></tr>
</table>

Table S4. Seasonal and diel patterns of bacterial and fungal α-diversity metrics under background sampling conditions.

Comparison	Domain	α-diversity metric	Group	Mean ± SD
Season	Bacteria	Shannon	Cold	4.254 ± 0.984
		Shannon	Warm	4.330 ± 0.824
		Chao1	Cold	443.17 ± 131.25
		Chao1	Warm	384.84 ± 121.65
	Fungi	Shannon	Cold	2.850 ± 0.489
		Shannon	Warm	3.003 ± 0.594
		Chao1	Cold	115.27 ± 72.48
		Chao1	Warm	142.81 ± 101.40
Diel	Bacteria	Shannon	Day	4.222 ± 1.003
		Shannon	Night	4.168 ± 0.933
		Chao1	Day	420.2 ± 128.7
		Chao1	Night	392.4 ± 119.9
	Fungi	Shannon	Day	2.813 ± 0.505
		Shannon	Night	3.098 ± 0.533

Comparison	Domain	α-diversity metric	Group	Mean ± SD
		Chao1	Day	114.7 ± 83.3
		Chao1	Night	148.0 ± 95.8

Table S5. Statistical tests for monthly and day–night differences in alpha- and beta-diversity indices.

Type	Index	Statistical test	Statistic-value	p value	q value
Bacteria (Cold/Warm)	Chao1	Mann-Whitney U	U=743.00	0.044	0.313
	Shannon	Mann-Whitney U	U=608.00	0.717	0.859
	Bray-Curtis	PERMANOVA	F=3.75	0.001	0.002
	-	PERMDISP	F=0.26	0.612	0.702
Fungi (Cold/Warm)	Chao1	Mann-Whitney U	U=516.00	0.451	0.751
	Shannon	Mann-Whitney U	U=462.00	0.157	0.313
	Bray-Curtis	PERMANOVA	F=10.11	0.001	0.002
	-	PERMDISP	F=0.04	0.833	0.833
Bacteria (Day/Night)	Chao1	Paired Wilcoxon	W=119.00	0.095	0.191
	Shannon	Paired Wilcoxon	W=160.00	0.500	0.500
	Bray-Curtis	Paired PERMANOVA	F=0.88	0.189	0.277*
	-	PERMDISP	F≈0.00	0.994	0.994*
Fungi (Day/Night)	Chao1	Paired Wilcoxon	W=101.00	0.034	0.034
	Shannon	Paired Wilcoxon	W=93.00	0.020	0.034
	Bray-Curtis	Paired PERMANOVA	F=3.29	0.001	0.004*
	-	PERMDISP	F=1.26	0.208	0.277*

Table S6 Monthly mean values of meteorological parameters and chemical components during the study period. Values summarize matched sampling months from November 2024 to November 2025. Units are shown in the variable names where applicable. The table provides the environmental background used to interpret seasonal community variation and downstream genus-environment association analyses.

Variable	2024-11	2025-01	2025-03	2025-05	2025-07	2025-09	2025-11
Temp (℃)	17.65	7.65	9.94	22.85	29.23	28.86	9.53
RH (%)	70.07	64.00	64.90	53.13	77.75	77.26	59.58
WS (m/s)	2.22	2.36	2.89	2.45	2.95	3.10	4.28
O_3 (μg/m^3)	72.67	45.93	72.14	126.1	30.88	87.24	34.62
NO_2 (μg/m^3)	28.90	48.93	30.90	20.68	9.64	19.07	46.64
$PM_{2.5}$ (μg/m^3)	29.24	31.40	30.40	23.77	14.77	19.32	30.41
WSIs (μg/m^3)	24.63	25.47	25.78	16.08	12.14	12.78	22.91
Metals (μg/m^3)	0.928	1.09	0.945	1.23	0.549	0.819	2.23

Table S7 Daily meteorological conditions and $PM_{2.5}$ chemical composition during the strong cold-air event in Shanghai, 16–20 November 2025.

Parameter	Unit	16 Nov	17 Nov	18 Nov	19 Nov	20 Nov
Minimum temperature	°C	14.4	5.6	4.2	3.5	1.9
Mean temperature	°C	19.1	12.4	6.4	6.6	7.9
Maximum temperature	°C	23.5	17.0	9.5	11.0	14.6
Mean relative humidity	%	70.9	71.4	41.8	43.3	63.2
Mean wind speed	$m\ s^{-1}$	2.62	7.65	5.98	3.96	1.71
Mean atmospheric pressure	hPa	1023.9	1030.6	1033.2	1030.5	1028.4
O_3	$\mu g\ m^{-3}$	55.5	37.6	30.8	27.4	21.8
NO_2	$\mu g\ m^{-3}$	43.8	22.7	32.8	54.2	79.6
SO_2	$\mu g\ m^{-3}$	6.6	6.7	9.1	8.7	11.7
CO	$mg\ m^{-3}$	0.53	0.68	0.75	0.83	1.09
SO_4^{2-}	$\mu g\ m^{-3}$	3.2	2.6	2.1	2.8	3.7
Cl^-	$\mu g\ m^{-3}$	1.3	1.2	1.6	1.7	1.8
NO_3^-	$\mu g\ m^{-3}$	5.183	3.333	8.543	14.543	21.245
NO_2^-	$\mu g\ m^{-3}$	0.393	0.496	0.117	0.387	0.377
Na^+	$\mu g\ m^{-3}$	0.619	0.471	0.273	0.404	0.140
NH_4^+	$\mu g\ m^{-3}$	4.438	3.595	5.181	6.982	9.087
K^+	$\mu g\ m^{-3}$	0.429	1.130	0.250	0.328	0.371
Mg^{2+}	$\mu g\ m^{-3}$	0.061	0.163	0.071	0.075	0.100

Parameter	Unit	16 Nov	17 Nov	18 Nov	19 Nov	20 Nov
Ca^{2+}	μg m^{-3}	0.633	1.107	0.886	0.593	0.551
SNA	μg m^{-3}	12.790	9.523	15.868	24.298	33.997
OC	μg m^{-3}	3.026	1.883	3.596	5.275	5.209
EC	μg m^{-3}	1.225	0.461	1.008	1.516	3.136
Al	μg m^{-3}	1.5616	0.9456	0.8702	0.8734	2.0159
Fe	μg m^{-3}	0.1995	0.2064	0.2388	0.2837	0.3309
Zn	μg m^{-3}	0.2552	0.1734	0.1552	0.2162	0.3894
Sb	μg m^{-3}	0.0983	0.0983	0.1377	0.1620	0.2170
Cu	μg m^{-3}	0.0737	0.0642	0.1525	0.1412	0.2215
Sr	μg m^{-3}	0.0826	0.0442	0.0387	0.0431	0.1109
Mn	μg m^{-3}	0.0270	0.0324	0.0345	0.0438	0.0539
Ba	μg m^{-3}	0.0282	0.0249	0.0313	0.0233	0.0331
Li	μg m^{-3}	0.0260	0.0152	0.0145	0.0202	0.0325
Pb	μg m^{-3}	0.0127	0.0133	0.0170	0.0290	0.0243
Ti	μg m^{-3}	0.0173	0.0162	0.0155	0.0157	0.0199
Cr	μg m^{-3}	0.0128	0.0101	0.0125	0.0098	0.0190
Ni	μg m^{-3}	0.0084	0.0091	0.0055	0.0079	0.0073
V	μg m^{-3}	0.0049	0.0054	0.0038	0.0040	0.0044
Mo	μg m^{-3}	0.0015	0.0014	0.0039	0.0032	0.0022
Cd	μg m^{-3}	0.0004	0.0003	0.0005	0.0007	0.0007
